# TIP_finder: An HPC Software to Detect Transposable Element Insertion Polymorphisms in Large Genomic Datasets

**DOI:** 10.3390/biology9090281

**Published:** 2020-09-09

**Authors:** Simon Orozco-Arias, Nicolas Tobon-Orozco, Johan S. Piña, Cristian Felipe Jiménez-Varón, Reinel Tabares-Soto, Romain Guyot

**Affiliations:** 1 Department of Computer Science, Universidad Autónoma de Manizales, Manizales 170002, Colombia; nicolas.tobono@autonoma.edu.co (N.T.-O.); johan.pinad@autonoma.edu.co (J.S.P.); 2 Department of Systems and Informatics, Universidad de Caldas, Manizales 170002, Colombia; 3 Department of Physics and Mathematics, Universidad Autónoma de Manizales, Manizales 170002, Colombia; cfjimenezv@gmail.com; 4 Department of Electronics and Automation, Universidad Autónoma de Manizales, Manizales 170002, Colombia; rtabares@autonoma.edu.co; 5 Institut de Recherche pour le Développement (IRD), CIRAD, Université de Montpellier, 34394 Montpellier, France

**Keywords:** TIP_finder, bioinformatics, HPC, parallel programming, polymorphism, HERV, post-genomic era, TIPs

## Abstract

Transposable elements (TEs) are non-static genomic units capable of moving indistinctly from one chromosomal location to another. Their insertion polymorphisms may cause beneficial mutations, such as the creation of new gene function, or deleterious in eukaryotes, e.g., different types of cancer in humans. A particular type of TE called LTR-retrotransposons comprises almost 8% of the human genome. Among LTR retrotransposons, human endogenous retroviruses (HERVs) bear structural and functional similarities to retroviruses. Several tools allow the detection of transposon insertion polymorphisms (TIPs) but fail to efficiently analyze large genomes or large datasets. Here, we developed a computational tool, named TIP_finder, able to detect mobile element insertions in very large genomes, through high-performance computing (HPC) and parallel programming, using the inference of discordant read pair analysis. TIP_finder inputs are (i) short pair reads such as those obtained by Illumina, (ii) a chromosome-level reference genome sequence, and (iii) a database of consensus TE sequences. The HPC strategy we propose adds scalability and provides a useful tool to analyze huge genomic datasets in a decent running time. TIP_finder accelerates the detection of transposon insertion polymorphisms (TIPs) by up to 55 times in breast cancer datasets and 46 times in cancer-free datasets compared to the fastest available algorithms. TIP_finder applies a validated strategy to find TIPs, accelerates the process through HPC, and addresses the issues of runtime for large-scale analyses in the post-genomic era.

## 1. Introduction

Transposable elements (TEs) are non-static genomic units capable of moving indistinctly from one chromosomal location to another [1,2,3]. These mobile elements can accumulate large copy numbers in their host genomes [4] and have been found in all organisms. The majority of the nuclear DNA content of large genomes is composed of TEs, such as in wheat, barley, and maize [5,6,7] for plants. In humans, these elements (or TE-derived sequences) comprise ~50–70% of the sequenced genome [8]. Several studies have indicated that TEs play crucial genomic roles involved in chromosome structuring, structural variation, the alteration of gene expression [5,7], evolution, the variation of genomic size, and environmental adaptation [9,10,11,12,13]. Nevertheless, these elements can also be associated with human diseases, such as different types of cancer [14,15,16]. TEs are classified into two major classes depending on their replication modes [17]. Accordingly, Class I or retrotransposons use an RNA molecule as an intermediate, while Class II or DNA transposons utilize a DNA intermediate. Each class can be hierarchically sub-classified into orders, super-families, lineages, and families [9,18,19]. Among retrotransposons, an order called LTR-retrotransposons bears structural and functional similarities to retroviruses, including the presence of long terminal repeats (LTR) at both ends that flank central coding domains and a similar replication cycle [20,21].

A particular kind of retrovirus present in the human genome is known as the human endogenous retrovirus (HERVs) and makes up approximatively 8% of the DNA sequenced [22]. HERVs carry genes (gag, pro, pol, and env) encoding essential proteins at the functional and structural levels [12,23]. These genes show overexpression in Mendelian diseases [24] and the etiology of cancer, including breast cancer [25,26], testicular cancer [27], melanoma [28], and germ cell tumors [29]. This overexpression could support the hypothesis that HERVs can be used in comparative studies to find polymorphisms caused by the insertion of these retroelements. Other TEs have been used in comparative studies to find transposon insertion polymorphisms (TIPs) associated with certain biological phenomena, such as the adaptation processes of rice [30], paramutation and gene silencing [31], and gene expression regulation in several organisms [32,33,34].

There are several computational tools to detect TIPs and most of them are based on the discordant location of read pairs and split reads [35,36], such as iTIS_PseTNC [37], Jitterbug [38], Transposeq [39], Metasv [40], DD_Detection [41], and TRACKPOSON [30]. The latter was the most recently developed pipeline for the detection of TIPs in gene pools and it was designed to unravel the transpositional activity of TEs in genomic datasets by applying a faster and validated methodology. Although the bioinformatic programs used by TRACKPOSON can be executed in multiple processors, this pipeline does not use any parallel strategies and as a consequence BLAST is executed in a non-efficient way and the BLAST output is processed serially (i.e., on one processor). In addition, this program cannot be run in more than one computational node (or computer) and has issues with the amount of disk space required. Thus, this pipeline is not scalable to current supercomputers (with hundreds of servers) and remains suboptimal for analyzing large genomes with a substantial amount of resequencing data, such as Arabica coffee with 1.3 Gb [42,43], maize (*Zea mays*) with 2.3 Gb [44], pine tree (*Pinus taeda*) with 22 billion base pairs [45], and the human genome with 3.3 Gb [46], as well as large datasets (e.g., 10K plant genomes [47] and the Earth BioGenome [48]) and huge databases such as the NCBI genome collection with sequence assemblies from more than 13,000 different organisms [49].

Therefore, it is important to take advantage of supercomputing, parallel programming [50], and high-performance computing (HPC) approaches to speed up the bioinformatics analyses of large genomes and huge datasets [51,52,53]. These techniques have been applied in several tasks, such as the hierarchical clustering of nucleotide sequences [54], scaled BLAST using CPU [52] or Xeon Phi [53,55] architectures, and a pipeline to analyze LTR-retrotransposons [2]. Message passing interface (MPI) [56] is a well-known library for parallel programming [57], which is used to run many sub-problems that are previously divided given different focuses [58].

Here, we present TIP_finder, a pipeline that can be applied to analyze large genomes and large resequencing datasets to discover TIPs using the classical methodology of inference of discordant read pairs as proposed by several pipelines. TIP_finder, written in python, proposes different BLAST engines (NCBI or MagicBLAST) and works under HPC techniques and parallel programming. It can be scaled on many computational nodes (or servers) and multi-core architectures, which makes it especially functional for applications in massive sequencing projects in the current (post) genomic era [24,34,59]. To test the performance of TIP_finder in a challenging way, we use the very large human genome and available breast cancer datasets, for detecting the insertion of HERVs in relation with this disease. In addition, we developed different utilities that automatically perform analyses, such as graphs over TIPs frequencies, peak charts, and statistical associations analyses related to a certain condition. TIP_finder version 1.0 is publicly available at https://github.com/simonorozcoarias/TIP_finder.

## 2. Materials and Methods

### 2.1. Implementation of TIP_finder

TIP_finder follows the strategy of the analysis of discordant read pair as proposed by several algorithms such as by [30] to detect TIPs using (i) short pair reads such as those obtained by Illumina, (ii) a reference genome sequence assembled at the chromosome level, and a database of consensus TE sequences (or other mobile elements like HERVs). With available programs, we identified several bottlenecks dramatically reducing the speed of the analysis and increasing the size of the output files such as: the execution time of the NCBI-BLAST program, the writing time and format of the BLAST results, the absence of parallelization or the non-use of a robust programming language.

**Figure 1:**
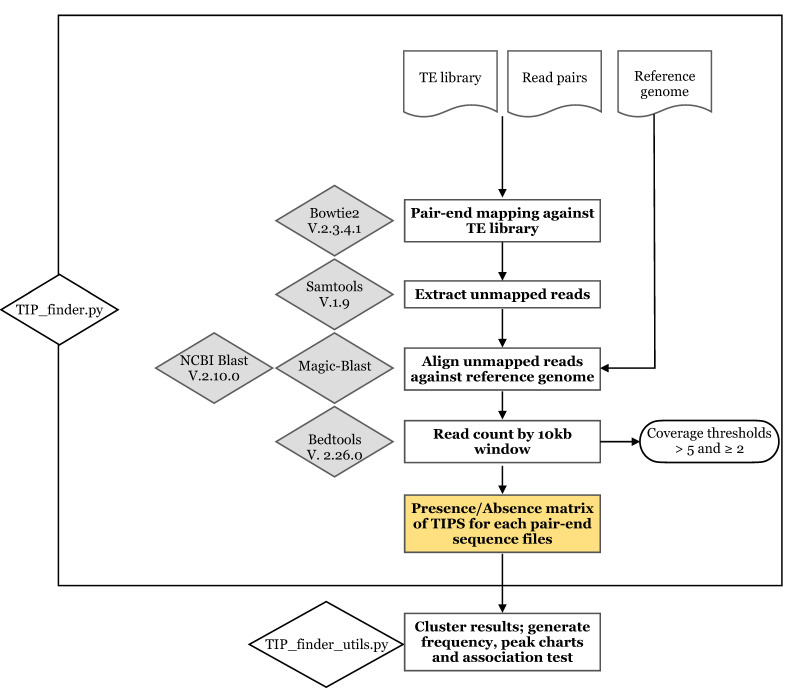
TIP_finder methodology and schematic representation of the pipeline. TE: transposable element, TIP: transposon insertion polymorphism.

The first step consisted of mapping paired reads to a mobile elements database and filtering only the reads showing one of the paired reads mapped. Then, the “unmapped reads” of read pairs are aligned to the reference genome to detect the insertion point of the putative TIP. The second step of TIP_finder processes the alignment output (saved as tabular format and only keeping the following columns: subject sequence id, start and end positions of the alignment in the subject sequence, and the ID of the query sequence) to search for reads with only one hit. This step produces a tabular file (in bed format) that will be used in the third step. Finally, TIP_finder counts how many reads were aligned to the reference genome, previously divided by a 10-Kb window, and generates a tabular-delimited file that is processed using the TIP_finder_utils.py. A general scheme of TIP_finder can be consulted in Figure 1.

TIP_finder_utils.py has four utilities to analyze and process the data generated, as follows: (1) cluster the results in a presence/absence (coded by 1/0) matrix of TIPs for each paired-end sequence file; (2) generate frequency graphs from the number of TIPs for each analyzed sample; (3) make peak charts using the frequency for TIPs present along the entire chromosome length; and (4) perform statistical association tests to identify TIPs displaying the highest association rate with a specific condition.

TIP_finder used the following bioinformatic software: Bowtie2 (v. 2.3.4.1) to map the paired reads of genomic data to the indexed consensus sequence of each TE/HERV family [30,60], Samtools (v. 1.9) to process the bowtie2 output and keep only the unmapped reads [61], bedtools (v. 2.26.0) to split the reference genome into 10 Kb windows and count reads in these windows [62], and NCBI-BLASTn (v. 2.10.0) [63] or Magic-BLAST [64] to align the unmapped reads. Although Magic-BLAST is significantly faster than NCBI-BLASTn, it requires more disk space. TIP_finder was developed using Python3 (3.8) and the following libraries: sys, time, os, subprocess, argparse, and MPI4py [65]. Furthermore, TIP_finder_utils.py requires the additional libraries: math, Pandas [66], matplotlib [67], Seaborn [68], and SciPy [69].

### 2.2. Parallel Strategy Implemented

**Figure 2:**
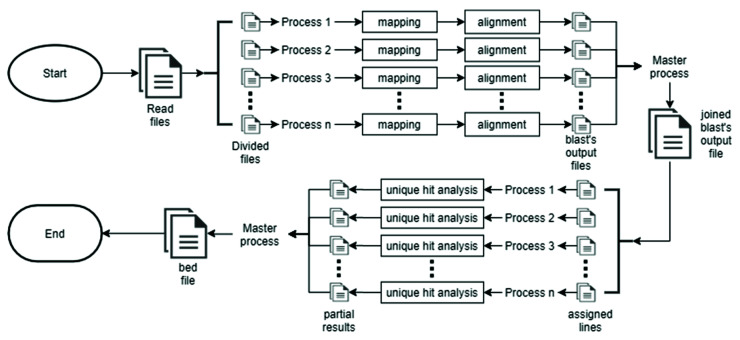
Flowchart of the parallel strategy implemented in TIP_finder.

To take advantage of multi-core architectures, TIP_finder is parallelized using the MPI standard and executes the same process several times with different data [51]. First, the read files (in FASTQ format) are split into n files (where n is the number of processes); then, each working process maps reads against the TE consensus databases. The mapping output is used to extract the “unmapped reads”, which are aligned against the reference genome. Each process generates a BLAST output file and the master process finally joins all of these into one file. The next step includes the detection of reads with a unique hit in the genome. For this, the number of lines in the BLAST output is divided by the processes number, and a start and end line number is assigned to each work process; the process analyzes its corresponding region and creates a Python dictionary, which is unified by the master process. Finally, each working process extracts reads with a unique hit and writes them to a file in bed format. The master process joins all the partial results, filters the final file, and deletes all the temporary files. An overall flowchart of this parallel strategy can be consulted in Figure 2.

### 2.3. Statistical Association Analysis

The statistical association tests performed using TIP_finder_utils.py consist of establishing two categorical random variables: X which determines if a patient *i* (*i*: 1,...,n) has a disease (cancer) and Y, which determines if a patient *i* has factor (TIP) *j* (*j*: 1,...,m). Given this, n corresponds to the number of patients (cases and controls) and m is the number of TIPs. The possible values associated with the aforementioned random variables are X = 0, 1 where X = 0 means that patient *i* does not have the disease and X = 1 means that the patient *i* has the disease, and Y = 0, 1 where Y = 0 means that patient *i* does not have the factor *j* and Y = 1 means that the patient *i* has factor *j* [70].

Based on a sample of n patients and m factors, the statistical association analysis is performed based on the chi-square distribution. Contingency tables are generated by crossing each of the m factors individually with the probability of having the disease or not. A model of the contingency table used is presented in Table 1.

The *O_ij_* values refer to the frequency observed in the data for patient *i* and the presence or absence of factor *j*.

Based on the values found in the contingency table, the totals by rows and columns are calculated to determine the expected frequency of each of the *O_ij_* observed frequencies. In order to determine whether factor *j* has a statistically significant influence on having the disease, we decided to propose a non-parametric independence hypothesis test based on the chi-square distribution. This test has the structure presented in (1) and (2) [71].

(1) $$H_{0}:\;Variables\;X\;and\;Y\;are\;independent.$$

(2) $$H_{1}:\;Variables\;X\;and\;Y\;are\;associated.$$

The chi-square statistics for these tests was calculated as follows:

(3) $$\chi^{2}{}_{Squared}=\overset{r}{\underset{i=1}{\sum }}\overset{s}{\underset{j=1}{\sum }}\frac{{\left(O_{ij}-e_{ij}\right)}^{2}}{e_{ij}},$$

where *O_ij_* is the observed frequency, *e_ij_* is the expected frequency, and *r*, *s* are the row and column sizes, respectively, which in this case are both worth two. The expected frequency *e_ij_* for each observed frequency *O_ij_* is calculated as follows:

(4) $$e_{ij}=\frac{n_{i.}n_{.j}}{n},$$

where $n_{i.}\;$refers to the total of row *i*, $n_{.j}$ refers to the total of column *j*, and n refers to the total of the joint data in the contingency table. Once the contrast statistic is determined $\chi^{2}{}_{calculated}$, a rejection criterion is proposed based on a significance level α and the degrees of freedom of the test given by FD=(r−1)(s−1). The criterion proposed is:

(5) $$\chi^{2}{}_{calculated}\geq \chi^{2}{}_{\alpha ,\left(r-1\right)\left(s-1\right)}.$$

The above test requires each of the expected frequencies to have a minimum value of five, in which case, the contrast statistic is recalculated by Yates’ correction for continuity. The new statistic is as follows:

(6) $$\chi^{2}{}_{calculated}=\sum_{i=1}^{r}\sum_{j=1}^{s}\frac{{\left(\left|O_{ij}-e_{ij}\right|-0.5\right)}^{2}}{e_{ij}}.$$

Finally, once the factors associated with having the disease are identified, we proceed to determine the value of the strength of the association, which ranges from 0 to 1 and is a measure of the degree of association found between variables X and Y. To do this, we calculated the contingency coefficient [72], which is estimated based on the chi-square statistic, as follows:

(7) $$C=\sqrt{\frac{\chi^{2}}{n+\chi^{2}}}.$$

### 2.4. Genomic Data Used by TIP_finder

We first use the coffee genome (*C. canephora*) [73] to test available tools. The pseudomolecule chr2 (with 53 Mb), was used as a reference genome, the LTR-retrotransposons (SIRE lineage) as the repeat library, and one resequencing Illumina data set for C. canephora (SRX5013724) [74].

In order to test the behavior and performance of TIP_finder, we used human endogenous retroviruses (HERVs) as a proof of concept since HERVs have been correlated with many tumors. For example, there are several studies reporting an overexpression of HERVs type K in several cancers, such as melanoma [28], prostate [27], pancreas [75], breast [26], and ovary [76]. Breast cancer was chosen here as a condition of interest [26] since it is the leading cause of death by cancer among women worldwide [77].

We used publicly available datasets from the NCBI Whole Genome Sequencing (WGS) database, corresponding to people with breast cancer (i.e., case patients) and people without the disease (i.e., control patients). Case patients were obtained from the SRA (Sequence Read Archive) database through search Equation (8). We retrieved 512 datasets and discarded those obtained by sequencing technologies other than Illumina and that lacked public permission for download. As a result, ~300 datasets of paired reads were pre-selected and only 10% were used (30 patients) due to the huge disk space required for storage. Control patients were consulted from Chen 2016 [78], obtaining a list of 30 patients from the BioProject PRJNA551447 on a study of schizophrenia in a Chinese population (Supplementary Material S1):

((((((((((breast cancer) AND Homo sapiens[Organism]) AND PAIRED[Layout])) AND GENOMIC[Source])) AND WGS[Strategy]))) NOT exome).(8)

For each patient (i.e., case and control), a maximum of 30 million reads was downloaded using the fastq-dump tool from the SRA toolkit [79]. Overall, the datasets comprised approximately 1.1 Terabytes of disk space (642 Gb for controls and 458 Gb for cases). HERV-K consensus sequences were obtained from Repbase (free version 2017) [80]. Finally, the human reference genome (3.3 Gb) in FASTA format was downloaded from the NCBI (assembly GRCh38.p13).

### 2.5. Computational Resources

TIP_finder was executed on a server with 56 cores E5-2695 v. 3, 252 GB RAM, and CentOS 7 managed by Slurm. Python 3 and the libraries used were installed using Anaconda 3 environments and pre-required software were loaded using environmental modules [81].

## 3. Results

### 3.1. Problems Encountered with Large Genomes and Testing TIP_finder

Several tools have been developed to search for transposable element insertions using the approach of discordant read pair analysis when mapped against a reference genome. Recently, we were interested in the study of the diversity of cultivated coffee trees (*C. canephora* and *C. arabica*) induced by the insertion of LTR-retrotransposons, the latter elements representing about 50% of the size of these genomes. The fastest and most accurate tool available (TRACKPOSON) was used with one family of LTR-retrotransposons (SIRE lineage) with a single set of resequencing data and a single pseudomolecule of *C. canephora* (representing 1/11 and 1/22 of the *C. canephora* and *C. arabica* pseudomolecules respectively). The result was obtained in 9474 seconds, and generated a BLAST output file of 7577.6 Mb. Several factors explain this result, such as bottlenecks in the execution time of the BLAST program, the writing time and format of the results, and the lack of overall parallelization of the tools. Based on this observation, we decided to develop a new tool that could overcome these different bottlenecks in data analysis time to obtain a faster program that could be more easily deployed on supercomputers.

Thus, TIP_finder was developed for use in current supercomputers to analyze TIPs in huge genomic datasets. We first tested TIP_finder on coffee data and we obtained a computational time of 594 seconds and a BLAST result file size of 448 Mb, showing a decrease in the execution time of 15.9X and a decrease in file size of 16.9X. To test TIP_finder on very large genomes, we decided to analyze the insertional polymorphisms caused by HERVs of type K in the human genome since several studies have demonstrated that these elements correlate with breast cancer [26], a disease that causes high mortality rates in women [77].

**Figure 3:**
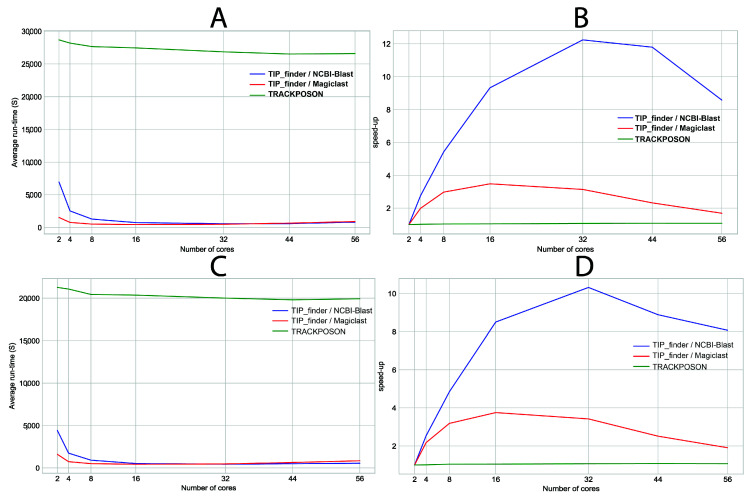
Total average runtime and speedup of TIP_finder -using NCBI-BLAST and MagicBLAST- and TRACKPOSON using 2, 4, 8, 16, 32, 44, and 56 cores with a randomly selected case dataset (30 million of reads) and executed 10 times (**A**,**B**), and a randomly selected control dataset (26.5 million of reads) and executed 10 times (**C**,**D**). The times for all executions can be found in Supplementary Material S2 Table S1 (for case dataset), and in Table S2 (for control dataset).

TIP_finder implements the MPI standard to reduce execution times; thus, allowing researchers to use more datasets. We tested TIP_finder (using NCBI-BLAST and MagicBLAST as aligners) and TRACKPOSON using a maximum of 30 million of reads (~1X of the human genome) from one randomly selected case dataset (Figure 3A,B) and one control dataset (Figure 3C,D) using 2, 4, 8, 16, 32, 44, and 56 cores, executed 10 times, to determine the behaviors of runtime (Figure 3A,C) and speedup (Figure 3B,D). To calculate the speedup, we used the execution with two cores as serial time since our MPI implementation used one processor as master and the others as workers.

**Figure 4:**
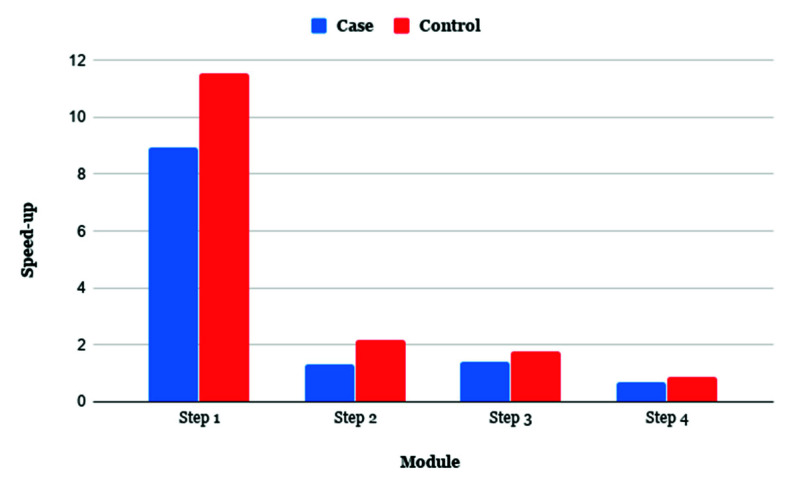
TIP_finder speedup of each step with 32 cores for one case and one control datasets.

We analyzed the behavior of TIP_finder for each of the four steps: Step 1: mapping and alignment, Step 2: creation of the dictionary with reads, Step 3: filter reads with one hit, and Step 4: the post-processing of TIPs. Here, we were particularly interested in monitoring the execution time and the speedup for each step with two different numbers of cores: two and 32. We used the same case and control datasets used for the speedup analyses. Figure 4 shows that Step 1 in both data sets benefited the most (up to 11.5X) from the parallel strategy used, because this Step is the most computationally time consuming (Figure S1).

**Figure 5:**
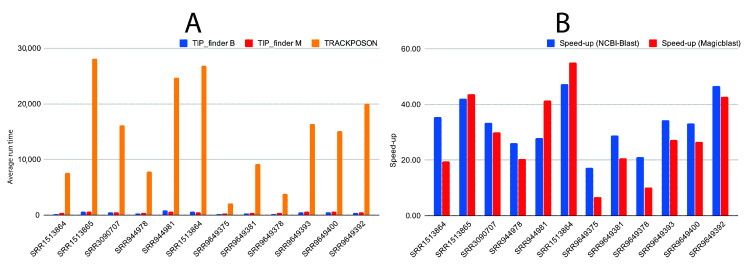
Total average runtime and speedup of TIP_finder and TRACKPOSON using six randomly selected control datasets and six randomly selected case datasets, each one executed 10 times. (**A**) Comparison of runtimes between TRACKPOSON and TIP_finder (with B: NCBI-BLAST, and M: Magic-BLAST), and (**B**) the comparison of the speedup of TIP_finder (NCBI-BLAST and MagicBLAST) compared to TRACKPOSON. Additional information can be consulted in Table S7.

Finally, we compared the execution times between TIP_finder and TRACKPOSON by randomly selecting five case and control datasets, in addition to the two patients used in the speedup analyses. This analysis aimed to measure the runtimes based on different numbers of TIPs in the datasets (Figure 5). All executions of TRACKPOSON and TIP_finder were performed using 32 cores and a maximum of 30 million reads.

### 3.2. Correlation of HERV-K TIPs with Breast Cancer as a Proof of Concept

After implementing TIP_finder and testing it in several configurations (e.g., different numbers of cores and datasets), we were interested in applying the software to a specific problem, such as the association of TIPs (in this case HERV-K) with breast cancer. We used publicly available Illumina read datasets found in the SRA database (NCBI) [82]. For this proof of concept, we selected 30 datasets for cases and 30 for controls. After obtaining the datasets, we ran TIP_finder to generate a tabular-separated file (in bed format) per individual, which contained the information of TIPs located throughout the chromosomes of each genome. Then, we ran TIP_finder_utils.py to automatically generate the analyses based on these data.

**Figure 6:**
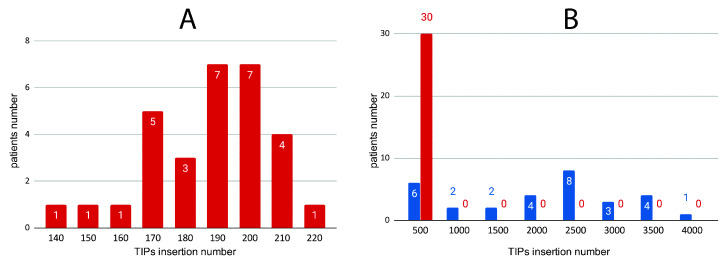
Number of TIPs insertions for the cases and controls identified by TIP_finder and grouped into bins of 500. Control and case patients are show in red, and in blue respectively. (**A**) Distribution of the number of TIPs insertions in cases and controls shown in blue and red, respectively. (**B**) Distribution of TIPs in controls within a range of 140–220.

TIP_finder_utils.py (“finalMatrix” utility) was run using the tabular-delimited file produced by TIP_finder.py to generate a presence/absence matrix of TIPs for each patient (using the bed file from TIP_finder produced for each individual). From this matrix, the number of TIPs for cases and controls were identified and grouped into bins. Figure 6A shows only the controls and displays the number of TIPs in a reduced bin with a specific range from 0 to 220. The cumulative number of TIPs in case patients, as seen in Figure 6B, is distributed over all bins (0–4000), while TIPs in control patients are only clustered into the first bin (0–500). These findings suggest a much higher insertional activity (HERV-K) for cases compared to controls (Figure 6).

**Figure 7:**
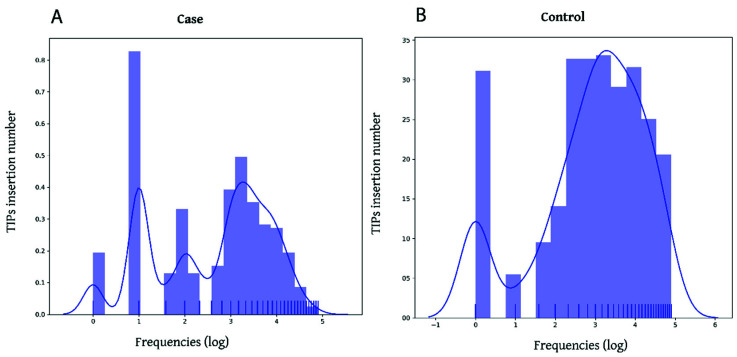
Distribution of TIPs insertions (Human endogenous retroviruses type K (HERV-K) insertions) for the cases and controls. The X axis represents the number of patients in log2 scale and the Y axis is the TIPs insertion number. (**A**,**B**) correspond to the cases and controls, respectively. Variations in the peaks’ frequency suggest a change in the insertional activity under a condition of interest, in this case cancer.

**Figure 8:**
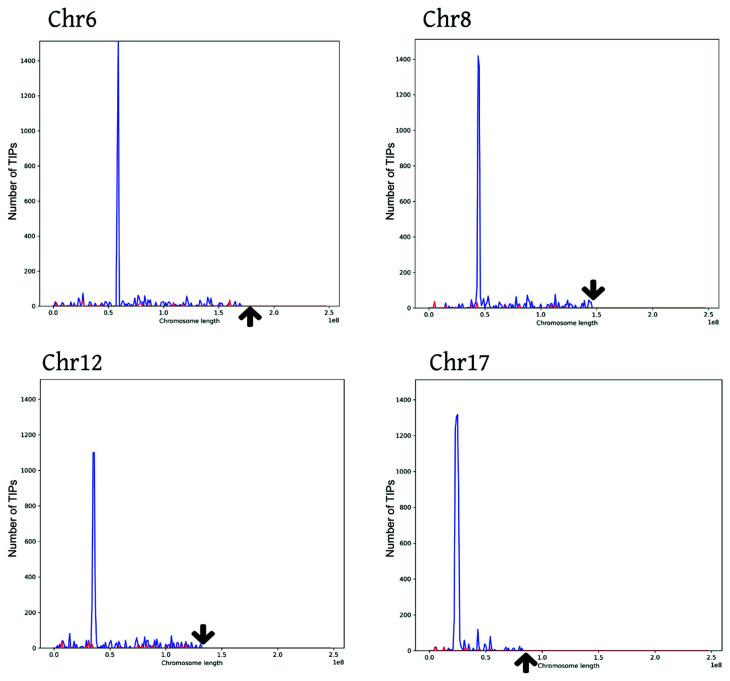
Distribution of the number of TIPs—HERVs—along human chromosomes. The X axis represents the chromosome length (on a scale of 1 × 10^8^), where cases and controls are shown in blue and red, respectively. The Y axis represents the number of TIPs along the chromosome length. The arrows show the end of each chromosome. The graphs for all chromosomes are available in Supplementary Material S4.

We executed the “histograms” utility of TIP_finder_utils.py to analyze the insertional activity of cases and controls. Figure 7A shows a high insertional activity of HERVs for case patients while control patients (Figure 7B) show a low insertion activity. Based on this insertional activity, the frequency of TIPs for both categories (cases and controls) were grouped according to their estimated position along each chromosome using bins of 300. TIP_finder split the reference genome into windows of 10 Kb, so the positions of each TIP were given within intervals of the window length. This clustering approach allowed detecting chromosome sections where the insertion of HERVs is markedly more frequent using the “peaks” utility of TIP_finder_utils.py. Figure 8 shows the distribution of TIPs for four of the 23 chromosomes (e.g., chromosomes 6, 8, 12, and 17), in which the peaks of polymorphic presence were most significant.

**Figure 9:**
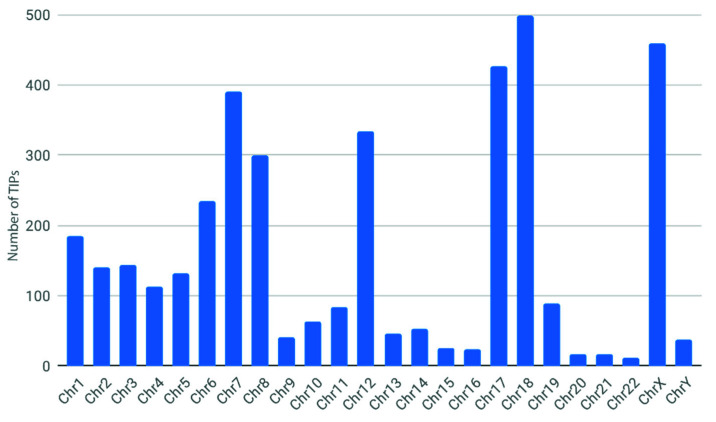
Number of TIPs statistically associated with breast cancer.

Finally, we performed a statistical association analysis through the “association” utility of TIP_finder_utils.py to investigate which TIPs may be associated with the condition of interest (i.e., breast cancer). We found a total of 3860 associated TIPs (significance level of 5%) along all the chromosomes. Chromosomes 18, X, 17, 7, 12, and 8 showed the highest associated TIPS with 498, 459, 427, 390, 233, and 299 TIPs, respectively (Figure 9 and Supplementary Material S3).

## 4. Discussion

The analysis of TIPs offers an efficient way to elucidate the dynamics of genome evolution in all organisms, from archaea to humans, via the activity of mobile elements. TIPs may help to answer multiple questions that arise in the field of genomics and bioinformatics, such as the process of the domestication of plants [30,82,83], how mobile elements have shaped the evolution of mammals [84], and inheritance features between organisms [85]. The study of genome diversity based on the insertional activity of TEs also provides an opportunity in the future to understand genetic diseases [24] caused by TIPs, such as breast cancer and its association with HERVs [26,86].

Although previous algorithms significantly improve the discovery of TIPs [30,87], their programming remains suboptimal considering that a parallel strategy and the use of multi-core architectures were not considered in their implementation. TRACKPOSON displays a speedup of ~1X (Figure 3) on 56 processors compared to two processors, due to the suboptimal execution of NCBI-BLAST and BLAST output processing using a single-processor Perl script. This demonstrates that a parallel strategy is required for better execution and this limitation is a disadvantage for massive sequencing projects that generate huge genomic datasets. To address this deficiency, the HPC and MPI techniques used by TIP_finder allow researchers to use many processors simultaneously in an efficient manner, taking advantage of all the benefits of supercomputers. TIP_finder can accelerate up to 55× the runtime compared to TRACKPOSON (Figure 3B,D). This improvement is important considering that TRACKPOSON is the fastest TIP detector in a recent benchmarking analysis [36]. Furthermore, TIP_finder can process larger datasets, improving its speedup using a high number of processors. Particularly, Step 1, which involves mapping (Bowtie2) and alignment (NCBI-BLAST or Magic-BLAST), requires the highest execution time and computational resources (Figure S1); thus, greatly benefiting from a parallel programming approach to speed up the analysis compared to TRACKPOSON (Figure 4). Furthermore, TIP_finder is a scalable software since it can run single-node applications (i.e., software than can run over multiple processors but are limited to one server), such as Bowtie2, NCBI-BLAST, and MagicBLAST, on multiple servers by splitting data into different inputs (i.e., data is split to run on different servers, see Figure 2). This feature allows taking advantage of current clusters that follow a distributed memory structure and makes the TIP_finder an especially useful tool for the massive sequencing projects required in the post-genomic era. Since TIP_finder applies a validated strategy to find TIPs [30], the results have high quality in terms of precision and sensitivity [36].

In a proof of concept using the sequencing data of 60 patients, TIP_finder demonstrated suitable performance for TIPs identification (represented here as hte consensus sequences of HERV-K) in the large human genome. Despite this not being the objective of the present study, the association between HERV-K and breast cancer in humans was confirmed. HERV-K in human breast cancer are involved in the processes of replication and tumorigenesis [88], derived directly from the protein and gene activities of mobile elements in the host [12]. This association is supported by the literature, indicating that this disease could be caused by the presence of HERVs in chromosomes 6, 7, 8, and 19 [26]. A larger sample of patients is necessary to further study this association and particularly, identify the coding regions involved. Moreover, TIP_finder should be tested on other human genetic diseases involving the mobility of endogenous retroviruses.

Overall, TIP_finder is a software that, besides its computational versatility (scalability and high performance), can run analyses on any group of organisms (plants, bacteria, animals) and uses information from any type of mobile element. Finally, this software will be used in future work on applications of the deep analysis of retrovirus interference or association with diseases in humans. These applications will help unravel potential applications in the industry of personalized medicine and pharmacology, as well as innovate with biological pathways for the treatment and prevention of the condition analyzed.

## 5. Conclusions

The development of HPC-based applications in bioinformatics is required on a daily basis. Thanks to the reduced cost of sequencing technologies, the challenge is not in obtaining genomic datasets, but rather in the analysis of this information in record time. Studies on TIPs have been performed in large datasets, demonstrating promising results for solving biological questions. Nevertheless, TIPs analyses show limitations in scalability since the available tools do not implement parallel strategies and follow a single-node approach. TIP_finder proves to be a very useful tool to be implemented on an HPC computing cluster architecture with many servers and with genomic datasets of considerable size. Moreover, the analysis of polymorphic activities of HERV-K could be used to perform a genomic-scale study of certain cancer types, such as breast cancer.

## Figures and Tables

**Table 1:** Contingency table used for the statistical association analysis.

Y/X	X = 0	X = 1
Y = 0	O_00_	O_01_
Y = 1	O_10_	O_11_

## Supplementary Materials

The following are available online at https://www.mdpi.com/2079-7737/9/9/281/s1, Supplementary Material S1: SRA (Sequence Read Archive) accessions list of the case and control patients used, Supplementary Material S2: Figure S1: The proportion of execution times per step using a randomly selected case dataset and: (A) two cores (serial) and (B) 32 cores; and utilizing a randomly selected control dataset and: (C) two cores (serial) and (D) 32 cores using NCBI-BLAST as aligner, Table S1: TIP_finder execution times using one randomly selected case dataset, Table S2: TIP_finder execution times using one randomly selected control dataset, Table S3: TIP_finder execution time per step using one randomly selected case dataset and two processors (serial), Table S4: TIP_finder execution time per step using one randomly selected case dataset and 32 processors, Table S5: TIP_finder execution time per step using one randomly selected control dataset and two processors (serial), Table S6: TIP_finder execution time per step using one randomly selected control dataset and 32 processors, Table S7: Comparative executions using TRACKPOSON and TIP_finder on five randomly selected case and control datasets and 32 cores, Supplementary Material S3: TIPs showing statistical association with breast cancer, Supplementary Material S4: Chromosomal distribution of TIPs in the human genome.
