# Supplementary material for: TIP_finder: An HPC Software to Detect Transposable Element Insertion Polymorphisms in Large Genomic Datasets
Source: Biology (Basel). 2020 Sep 9;9(9):281. doi: 10.3390/biology9090281 (PMC7563458; doi:10.3390/biology9090281)
Supplement: Supplementary file 1 [file biology-09-00281-s001.zip › Supplemental Material 1.docx]

| **SRA Accession List - Cases** | | | | |
| --- | --- | --- | --- | --- |
| **Accession** | **# of Spots** | **#of Bases** | **Size** | **Study or BioProject** |
| ERR232239 | 21,851,875 | 3.3G | 1.6Gb | [Barber LJ *et al.,*](https://www.ncbi.nlm.nih.gov/pubmed/21750719) "Comprehensive genomic analysis of a BRCA2 deficient human pancreatic cancer.", *PLoS One*, 2011;6(7):e21639 |
| ERR232240 | 23,189,082 | 3.5G | 1.7Gb |  |
| ERR232241 | 22,981,389 | 3.5G | 1.7Gb |  |
| ERR232242 | 22,621,836 | 3.4G | 1.6Gb |  |
| ERR232243 | 22,639,118 | 3.4G | 1.6Gb |  |
| ERR232244 | 22,261,938 | 3.4G | 1.5Gb |  |
| ERR232245 | 21,527,631 | 3.3G | 1.4Gb |  |
| ERR232246 | 10,516,200 | 1.6G | 1.1Gb |  |
| ERR232247 | 10,430,921 | 1.6G | 1.1Gb |  |
| ERR232248 | 10,198,798 | 1.6G | 1.1Gb |  |
| ERR232249 | 9,719,346 | 1.5G | 1.1Gb |  |
| ERR232250 | 9,473,836 | 1.4G | 1Gb |  |
| ERR232251 | 9,749,436 | 1.5G | 1.1Gb |  |
| ERR232252 | 9,571,910 | 1.5G | 1Gb |  |
| SRR1513864 | 176,642,889 | 35.3G | 19.7Gb | [**PRJNA253369**](https://www.ncbi.nlm.nih.gov/bioproject/PRJNA253369) |
| SRR1513865 | 133,306,847 | 26.7G | 15.3Gb |  |
| SRR3090651 | 60,374,706 | 11.8G | 7.5Gb | [**PRJNA308098**](https://www.ncbi.nlm.nih.gov/bioproject/PRJNA308098) |
| SRR3090701 | 67,475,052 | 13.2G | 8.5Gb |  |
| SRR3090702 | 67,950,524 | 13.3G | 8.6Gb |  |
| SRR3090705 | 53,908,355 | 10.6G | 6.8Gb |  |
| SRR3090707 | 37,496,500 | 7.3G | 4.7Gb |  |
| SRR3090723 | 43,821,190 | 8.6G | 5.5Gb |  |
| SRR944977 | 184,296,718 | 36G | 21.3Gb | [**PRJNA213134**](https://www.ncbi.nlm.nih.gov/bioproject/PRJNA213134) |
| SRR944978 | 181,844,287 | 36.4G | 21.6Gb |  |
| SRR944979 | 182,902,360 | 36.6G | 21.8Gb |  |
| SRR944980 | 181,022,543 | 36.2G | 21.5Gb |  |
| SRR944981 | 181,130,440 | 36.2G | 21.5Gb |  |
| SRR944982 | 185,812,676 | 37.2G | 22.1Gb |  |
| SRR944983 | 182,795,197 | 36.6G | 21.7Gb |  |
| SRR944984 | 182,335,586 | 36.5G | 21.6Gb |  |
| **SRA Accession list - controls** | | | | |
| **Accession** | **# of Spots** | **#of Bases** | **Size** | **Study or BioProject** |
| SRR9649373 | 370,350,202 | 111.1G | 44.5Gb | [PRJNA551447](https://www.ncbi.nlm.nih.gov/bioproject/PRJNA551447) |
| SRR9649374 | 331,534,072 | 99.5G | 39.4Gb |  |
| SRR9649375 | 331,534,072 | 99.5G | 39.4Gb |  |
| SRR9649376 | 311,053,284 | 93.3G | 39.4Gb |  |
| SRR9649377 | 301,783,741 | 90.5G | 38.6Gb |  |
| SRR9649378 | 298,399,259 | 89.5G | 37.8Gb |  |
| SRR9649379 | 322,309,329 | 96.7G | 40.3Gb |  |
| SRR9649380 | 326,703,844 | 98G | 41.5Gb |  |
| SRR9649381 | 319,430,885 | 95.8G | 40.6Gb |  |
| SRR9649382 | 313,457,184 | 94G | 38.8Gb |  |
| SRR9649383 | 316,140,982 | 94.8G | 38.8Gb |  |
| SRR9649384 | 353,893,374 | 106.2G | 45Gb |  |
| SRR9649385 | 378,484,273 | 113.5G | 47.7Gb |  |
| SRR9649386 | 340,592,921 | 102.2G | 42.6Gb |  |
| SRR9649387 | 321,505,765 | 96.5G | 39.8Gb |  |
| SRR9649388 | 361,660,610 | 108.5G | 43.2Gb |  |
| SRR9649389 | 388,987,793 | 116.7G | 46.1Gb |  |
| SRR9649390 | 327,405,862 | 98.2G | 41.4Gb |  |
| SRR9649391 | 316,923,845 | 95.1G | 39.5Gb |  |
| SRR9649392 | 348,472,163 | 104.5G | 43.6Gb |  |
| SRR9649393 | 357,743,831 | 107.3G | 42.5Gb |  |
| SRR9649394 | 315,378,602 | 94.6G | 38.6Gb |  |
| SRR9649395 | 817,072,486 | 122.6G | 58.8Gb |  |
| SRR9649396 | 303,008,395 | 90.9G | 38.3Gb |  |
| SRR9649397 | 340,865,126 | 102.3G | 42.9Gb |  |
| SRR9649398 | 385,029,280 | 115.5G | 47.5Gb |  |
| SRR9649399 | 314,613,420 | 94.4G | 41.4Gb |  |
| SRR9649400 | 310,583,203 | 93.2G | 39.4Gb |  |
| SRR9649401 | 339,111,152 | 101.7G | 43.9Gb |  |
| SRR9649402 | 338,916,948 | 101.7G | 41.6Gb |  |
| SRR9649403 | 327,357,465 | 98.2G | 42.4Gb |  |
