# Supplementary material for: TIP_finder: An HPC Software to Detect Transposable Element Insertion Polymorphisms in Large Genomic Datasets
Source: Biology (Basel). 2020 Sep 9;9(9):281. doi: 10.3390/biology9090281 (PMC7563458; doi:10.3390/biology9090281)
Supplement: Supplementary file 1 [file biology-09-00281-s001.zip › Supplemental Material 2.docx]

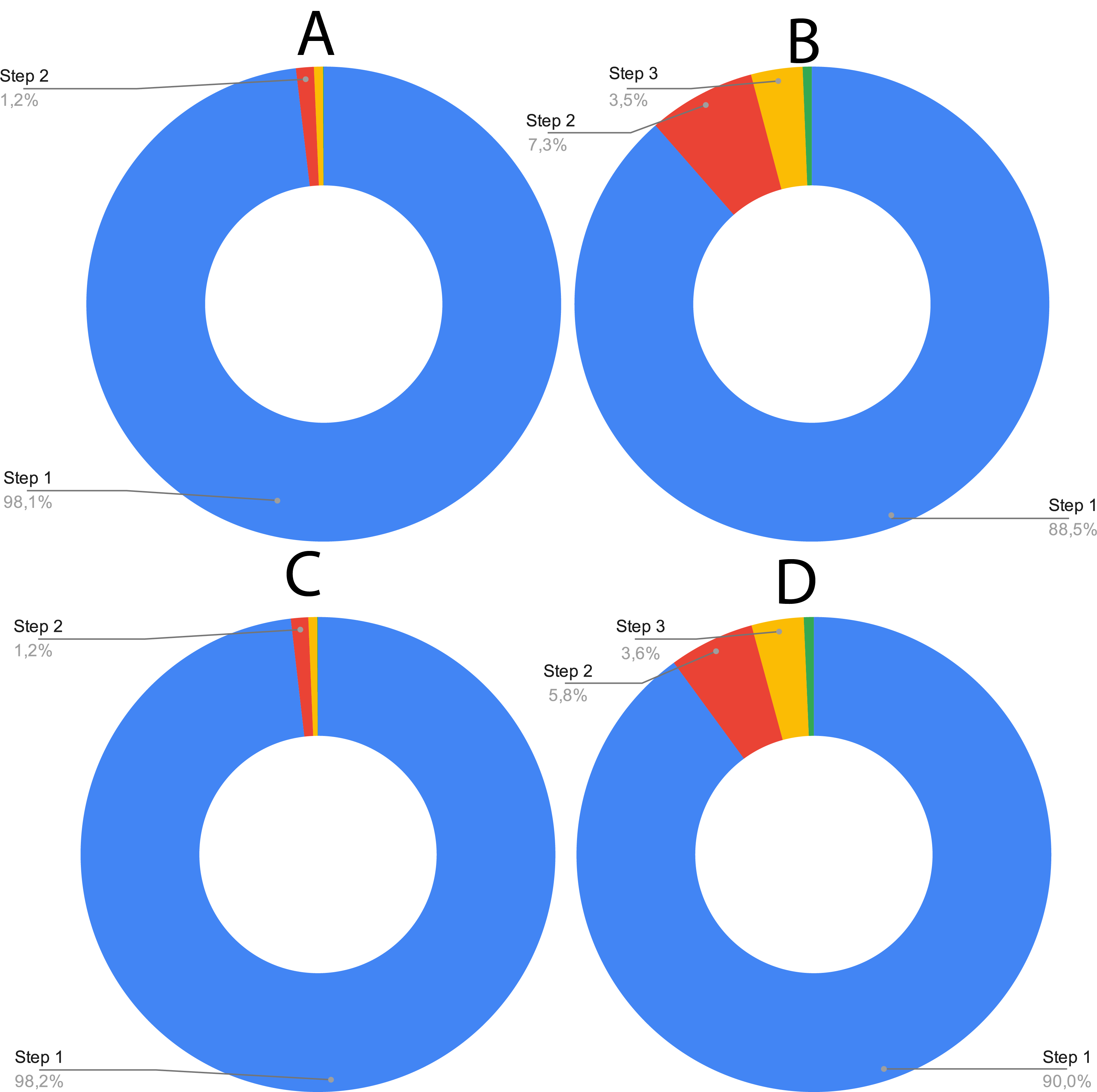


Figure S1. Proportion of execution times per step using a randomly selected case dataset and: A) two cores (serial) and B) 32 cores; and utilizing a randomly selected control dataset and: C) two cores (serial) and D) 32 cores using NCBI-Blast as aligner. The steps not shown correspond to less than 1% of the total execution time. Step 1: mapping and alignment, step 2: creation of dictionary with reads, step 3: filter reads with one hit, and step 4: post-processing TIPs. The execution times for each step can be found in Tables S3 and S4 (for case dataset), and in Tables S5 and S6 (for control dataset).

Table S1. TIP_finder Execution times using one randomly selected case dataset.

| TIP_finder (NCBI-Blast | | | | | | | | | | | | | | | | | | | | | |
| --- | --- | --- | --- | --- | --- | --- | --- | --- | --- | --- | --- | --- | --- | --- | --- | --- | --- | --- | --- | --- | --- |
| Cores | 1 | 2 | 3 | 4 | | 5 | | 6 | 7 | | 8 | | 9 | 10 | | | Average | | STD | | Speed up |
| 2 | 7410 | 7358 | 7146 | 6623 | | 6823 | | 6789 | 7048 | | 6854 | | 6789 | 6639 | | | 6947,9 | | 280,253 | | 1 |
| 4 | 2586 | 2385 | 2567 | 2646 | | 2590 | | 2470 | 2412 | | 2614 | | 2542 | 2484 | | | 2529,6 | | 87,828 | | 2,75 |
| 8 | 1279 | 1343 | 1291 | 1262 | | 1253 | | 1274 | 1270 | | 1259 | | 1299 | 1263 | | | 1279,3 | | 26,596 | | 5,43 |
| 16 | 714 | 957 | 715 | 718 | | 730 | | 741 | 713 | | 707 | | 729 | 725 | | | 744,9 | | 75,203 | | 9,33 |
| 32 | 552 | 582 | 601 | 523 | | 596 | | 544 | 564 | | 554 | | 589 | 573 | | | 567,8 | | 24,952 | | 12,24 |
| 44 | 607 | 598 | 604 | 595 | | 601 | | 595 | 589 | | 571 | | 567 | 564 | | | 589,1 | | 15,913 | | 11,79 |
| 56 | 812 | 808 | 809 | 814 | | 811 | | 805 | 806 | | 825 | | 800 | 808 | | | 809,8 | | 6,630 | | 8,58 |
| TIP_finder (Magicblast) | | | | | | | | | | | | | | | | | | | | | |
| Cores | 1 | 2 | 3 | 4 | 5 | | 6 | | | 7 | | 8 | 9 | | 10 | Average | | STD | | Speed-up | |
| 2 | 1671 | 1651 | 1534 | 1500 | 1469 | | 1462 | | | 1474 | | 1489 | 1483 | | 1576 | 1530,9 | | 76,679 | | 1 | |
| 4 | 737 | 777 | 754 | 805 | 783 | | 778 | | | 753 | | 753 | 792 | | 776 | 770,8 | | 20,933 | | 1,99 | |
| 8 | 562 | 532 | 495 | 493 | 492 | | 528 | | | 518 | | 493 | 497 | | 539 | 514,9 | | 24,660 | | 2,97 | |
| 16 | 595 | 423 | 423 | 425 | 424 | | 429 | | | 420 | | 427 | 419 | | 417 | 440,2 | | 54,512 | | 3,48 | |
| 32 | 564 | 504 | 483 | 467 | 475 | | 478 | | | 489 | | 482 | 472 | | 466 | 488 | | 28,952 | | 3,14 | |
| 44 | 764 | 634 | 630 | 641 | 663 | | 661 | | | 664 | | 667 | 640 | | 635 | 659,9 | | 39,233 | | 2,32 | |
| 56 | 1042 | 882 | 899 | 892 | 875 | | 889 | | | 898 | | 910 | 883 | | 890 | 906 | | 48,799 | | 1,69 | |
| TRACKPOSON | | | | | | | | | | | | | | | | | | | | | |
| Cores | 1 | 2 | 3 | 4 | | 5 | | 6 | 7 | | 8 | | 9 | 10 | | | Average | | STD | | Speed-up |
| 2 | 29093 | 28375 | 28534 | 28644 | | 28744 | | 28579 | 28615 | | 28531 | | 28824 | 28856 | | | 28679,50 | | 205,11 | | 1,00 |
| 4 | 28532 | 28290 | 27972 | 28015 | | 28238 | | 28228 | 28082 | | 28281 | | 28185 | 28080 | | | 28190,30 | | 163,62 | | 1,02 |
| 8 | 27730 | 27800 | 27729 | 27714 | | 27352 | | 27693 | 27473 | | 27570 | | 27729 | 27782 | | | 27657,20 | | 145,59 | | 1,04 |
| 16 | 27089 | 27040 | 27423 | 27727 | | 27650 | | 27396 | 27804 | | 27687 | | 27243 | 27486 | | | 27454,50 | | 267,54 | | 1,04 |
| 32 | 27075 | 26602 | 26730 | 27110 | | 26698 | | 26778 | 27038 | | 26924 | | 26629 | 26955 | | | 26853,90 | | 189,43 | | 1,07 |
| 44 | 26939 | 26320 | 26473 | 26470 | | 26315 | | 26758 | 26601 | | 26404 | | 26627 | 26337 | | | 26524,40 | | 206,48 | | 1,08 |
| 56 | 26547 | 26873 | 26121 | 26276 | | 26819 | | 26704 | 26858 | | 26693 | | 26143 | 26858 | | | 26589,20 | | 301,84 | | 1,08 |

Table S2. TIP_finder Execution times using one randomly selected control dataset.

| TIP_finder (NCBI-Blast) | | | | | | | | | | | | | |
| --- | --- | --- | --- | --- | --- | --- | --- | --- | --- | --- | --- | --- | --- |
| Cores | 1 | 2 | 3 | 4 | 5 | 6 | 7 | 8 | 9 | 10 | Average | STD | Speed-up |
| 2 | 4624 | 4830 | 4667 | 4374 | 4224 | 4337 | 4252 | 4464 | 4212 | 4245 | 4422,9 | 216,567 | 1 |
| 4 | 1989 | 1807 | 1857 | 1727 | 1777 | 1641 | 1609 | 1594 | 1757 | 1653 | 1741,1 | 123,756 | 2,54 |
| 8 | 917 | 925 | 925 | 906 | 917 | 912 | 915 | 890 | 906 | 913 | 912,6 | 10,276 | 4,85 |
| 16 | 657 | 506 | 506 | 508 | 496 | 510 | 506 | 502 | 503 | 508 | 520,2 | 48,228 | 8,50 |
| 32 | 435 | 432 | 425 | 447 | 408 | 422 | 422 | 430 | 435 | 430 | 428,6 | 10,330 | 10,32 |
| 44 | 498 | 507 | 497 | 511 | 497 | 489 | 491 | 495 | 496 | 496 | 497,7 | 6,651 | 8,89 |
| 56 | 547 | 549 | 547 | 547 | 547 | 551 | 544 | 549 | 549 | 550 | 548 | 2,000 | 8,07 |
| TIP_finder (Magicblast) | | | | | | | | | | | | | |
| Cores | 1 | 2 | 3 | 4 | 5 | 6 | 7 | 8 | 9 | 10 | Average | STD | Speed-up |
| 2 | 1681 | 1706 | 1623 | 1567 | 1485 | 1444 | 1438 | 1573 | 1708 | 1802 | 1602,70 | 123,08 | 1,00 |
| 4 | 787 | 748 | 751 | 770 | 759 | 736 | 677 | 727 | 686 | 719 | 736,00 | 34,97 | 2,18 |
| 8 | 587 | 495 | 479 | 511 | 502 | 500 | 492 | 488 | 484 | 494 | 503,20 | 30,84 | 3,19 |
| 16 | 525 | 393 | 395 | 396 | 389 | 408 | 400 | 431 | 484 | 449 | 427,00 | 45,98 | 3,75 |
| 32 | 454 | 459 | 472 | 463 | 462 | 504 | 445 | 462 | 442 | 521 | 468,40 | 25,16 | 3,42 |
| 44 | 764 | 635 | 629 | 636 | 615 | 606 | 617 | 601 | 642 | 630 | 637,50 | 46,44 | 2,51 |
| 56 | 987 | 829 | 834 | 811 | 841 | 802 | 819 | 841 | 815 | 830 | 840,90 | 52,93 | 1,91 |
| TRACKPOSON | | | | | | | | | | | | | |
| Cores | 1 | 2 | 3 | 4 | 5 | 6 | 7 | 8 | 9 | 10 | Average | STD | Speed-up |
| 2 | 21242 | 21150 | 21375 | 21403 | 21582 | 20944 | 21406 | 21402 | 21276 | 20875 | 21265,50 | 220,93 | 1,00 |
| 4 | 21368 | 21311 | 21248 | 21268 | 20760 | 21122 | 20750 | 20879 | 20767 | 21248 | 21072,10 | 253,62 | 1,01 |
| 8 | 20574 | 20605 | 20716 | 20612 | 20816 | 20399 | 20071 | 20268 | 20130 | 20167 | 20435,80 | 264,62 | 1,04 |
| 16 | 20154 | 20207 | 19857 | 20433 | 20464 | 20511 | 20454 | 20458 | 20544 | 20491 | 20357,30 | 217,86 | 1,04 |
| 32 | 20118 | 20189 | 20343 | 20089 | 19782 | 19931 | 19776 | 20181 | 19772 | 19843 | 20002,40 | 207,37 | 1,06 |
| 44 | 19803 | 19853 | 19916 | 19807 | 19806 | 19896 | 19730 | 19669 | 19892 | 19607 | 19797,90 | 101,82 | 1,07 |
| 56 | 19999 | 19968 | 19936 | 19889 | 19643 | 19960 | 19957 | 20131 | 19860 | 19887 | 19923,00 | 124,09 | 1,07 |

Table S3. TIP_finder execution time per step using one randomly selected case dataset and two processors (serial).

| **Module** | **1** | **2** | **3** | **4** | **5** | **6** | **7** | **8** | **9** | **10** | **Average** | **STD** | **Proportion** |
| --- | --- | --- | --- | --- | --- | --- | --- | --- | --- | --- | --- | --- | --- |
| Mapping and blast | 6.652,65 | 4.307,34 | 4.155,16 | 4.110,78 | 4.073,12 | 4.118,42 | 4.251,47 | 4.149,48 | 4.082,35 | 4.097,31 | 4.399,81 | 795,13 | 98,12 |
| create dictionary with reads | 82,91 | 45,57 | 53,49 | 51,76 | 53,21 | 52,74 | 51,65 | 51,30 | 51,77 | 51,30 | 54,57 | 10,20 | 1,22 |
| filter reads with one hit | 41,65 | 29,04 | 25,42 | 25,48 | 25,24 | 25,32 | 25,37 | 25,36 | 26,34 | 25,28 | 27,45 | 5,12 | 0,61 |
| post-processing TIPs | 2,87 | 2,33 | 2,34 | 2,21 | 2,52 | 2,20 | 2,80 | 2,69 | 2,47 | 2,30 | 2,47 | 0,24 | 0,06 |

Table S4. TIP_finder execution time per step using one randomly selected case dataset and 32 processors.

| **Module** | **1** | **2** | **3** | **4** | **5** | **6** | **7** | **8** | **9** | **10** | **Average** | **STD** | **Speed-up** | **Proportion** |
| --- | --- | --- | --- | --- | --- | --- | --- | --- | --- | --- | --- | --- | --- | --- |
| Mapping and blast | 476,64 | 507,05 | 525,78 | 446,63 | 519,12 | 467,75 | 490,95 | 483,18 | 512,04 | 501,01 | 493,02 | 24,80 | 8,92 | 88,55 |
| create dictionary with reads | 42,26 | 40,14 | 40,65 | 40,74 | 41,65 | 41,51 | 40,29 | 38,67 | 42,32 | 39,27 | 40,75 | 1,21 | 1,34 | 7,32 |
| filter reads with one hit | 19,03 | 20,04 | 20,52 | 20,55 | 21,46 | 20,19 | 18,34 | 17,24 | 19,30 | 18,76 | 19,54 | 1,25 | 1,40 | 3,51 |
| post-processing TIPs | 3,34 | 3,57 | 3,62 | 3,49 | 3,44 | 3,32 | 3,49 | 3,40 | 3,72 | 3,51 | 3,49 | 0,12 | 0,71 | 0,63 |

Table S5. TIP_finder execution time per step using one randomly selected control dataset and two processors (serial).

| **Module** | **1** | **2** | **3** | **4** | **5** | **6** | **7** | **8** | **9** | **10** | **Average** | **STD** | **Proportion** |
| --- | --- | --- | --- | --- | --- | --- | --- | --- | --- | --- | --- | --- | --- |
| Mapping and blast | 5059,35 | 4741,96 | 4569,74 | 4163,91 | 4286,54 | 4136,16 | 4249,30 | 4374,42 | 4122,67 | 4156,68 | 4386,07 | 312,03 | 98,16 |
| create dictionary with reads | 59,93 | 47,71 | 57,19 | 51,17 | 52,55 | 52,16 | 51,76 | 51,65 | 51,79 | 52,10 | 52,80 | 3,39 | 1,18 |
| filter reads with one hit | 31,21 | 30,12 | 28,71 | 25,62 | 25,45 | 25,39 | 25,72 | 25,19 | 25,03 | 25,61 | 26,81 | 2,30 | 0,60 |
| post-processing TIPs | 2,87 | 2,43 | 2,52 | 2,67 | 2,19 | 2,53 | 2,48 | 2,55 | 2,65 | 2,52 | 2,54 | 0,18 | 0,06 |

Table S6. TIP_finder execution time per step using one randomly selected control dataset and 32 processors.

| Module | 1 | 2 | 3 | 4 | 5 | 6 | 7 | 8 | 9 | 10 | Average | STD | Control | Proportion |
| --- | --- | --- | --- | --- | --- | --- | --- | --- | --- | --- | --- | --- | --- | --- |
| Mapping and blast | 375,04 | 378,41 | 393,44 | 395,40 | 378,49 | 371,75 | 379,28 | 376,20 | 376,79 | 380,86 | 380,57 | 7,73 | 11,53 | 89,95 |
| create dictionary with reads | 24,25 | 26,65 | 23,43 | 24,67 | 24,89 | 23,62 | 24,96 | 24,53 | 24,29 | 24,47 | 24,58 | 0,88 | 2,15 | 5,81 |
| filter reads with one hit | 14,43 | 15,60 | 14,46 | 15,84 | 14,03 | 15,05 | 16,07 | 15,08 | 14,29 | 15,37 | 15,02 | 0,70 | 1,78 | 3,55 |
| post-processing TIPs | 3,04 | 2,87 | 2,70 | 2,69 | 3,02 | 2,75 | 3,19 | 3,30 | 2,76 | 2,77 | 2,91 | 0,22 | 0,87 | 0,69 |

Table S7. Comparative executions using TRACKPOSON and TIP_finder in five randomly selected case and control datasets and 32 cores.

|  | Cases | | | | | | Controls | | | | | |
| --- | --- | --- | --- | --- | --- | --- | --- | --- | --- | --- | --- | --- |
|  | SRR1513864 | SRR1513865 | SRR3090707 | SRR944978 | SRR944981 | SRR1513864 | SRR9649375 | SRR9649381 | SRR9649378 | SRR9649393 | SRR9649400 | SRR9649392 |
| Reads (M) | 7,5 | 24,5 | 21,9 | 7,8 | 24,3 | 30 | 3,1 | 12,5 | 7,1 | 21,6 | 21,4 | 26,5 |
| TIPs Candidates | 1526 | 5592 | 3376 | 107 | 601 | 6502 | 39 | 198 | 77 | 480 | 334 | 505 |
| TIPs Confimed* | 100 | 1591 | 1142 | 2 | 25 | 2281 | 1 | 10 | 2 | 35 | 16 | 39 |
| TIP_finder B | 215 | 668 | 483 | 298 | 883 | 567 | 121 | 320 | 181 | 476 | 456 | 428 |
| TIP_finder M | 390 | 644 | 540 | 382 | 598 | 488 | 315 | 447 | 379 | 601 | 571 | 468 |
| TRACKPOSON | 7614 | 28098 | 16166 | 7814 | 24750 | 26861 | 2085 | 9216 | 3828 | 16383 | 15138 | 20003 |
| Speed-up (NCBI-Blast) | 35,41 | 42,06 | 33,47 | 26,22 | 28,03 | 47,37 | 17,23 | 28,80 | 21,15 | 34,42 | 33,20 | 46,74 |
| Speed-up (Magicblast) | 19,52 | 43,63 | 29,94 | 20,46 | 41,39 | 55,04 | 6,62 | 20,62 | 10,10 | 27,26 | 26,51 | 42,74 |
